# Subtle QRS changes are associated with reduced ejection fraction, diastolic dysfunction, and heart failure development and therapy responsiveness: Applications for artificial intelligence to ECG

**DOI:** 10.1111/anec.12998

**Published:** 2022-07-29

**Authors:** Artemio García‐Escobar, Silvio Vera‐Vera, Alfonso Jurado‐Román, Santiago Jiménez‐Valero, Guillermo Galeote, Raúl Moreno

**Affiliations:** ^1^ Cardiology Department, Interventional Cardiology Section La Paz University Hospital Madrid Spain; ^2^ Institute for Health Research La Paz University Hospital (IdiPAZ) Madrid Spain; ^3^ Biomedical Research Network Center on Cardiovascular Disease Institute of Health Carlos III Madrid Spain

**Keywords:** artificial intelligence, QRS axis, QRS duration, QRS fragmentation, QRS voltage, QT interval

## Abstract

**Background:**

Since the last century, the electrocardiogram (ECG) remains the non‐invasive test, that is, most easily accessible, feasible, and inexpensive for cardiology assessment. In past years, many novel ECG indexes and patterns have been published that allow for a more advanced evaluation of what is currently being done, especially based on subtle QRS changes and patterns.

**Objective:**

The objective of the study was to provide an update on the evidence and clinical applications of these ECG subtle QRS changes and patterns associated with heart disease.

**Methods:**

Through the literature review, we will highlight the subtle QRS changes and patterns associated with heart disease, mainly focusing on QRS duration, voltage, morphology, axis, and QT interval.

**Results:**

Small increases in QRS duration are associated with a reduction in left ventricular ejection fraction (EF), increased cardiac chamber dimensions, and risk for incident heart failure (HF). Moreover, fragmentation of the QRS complex is associated with myocardial fibrosis and is a substrate for developing arrhythmic events. Besides, low amplitude QRS voltage is associated with congestive HF, and an increase in the voltage of the QRS complexes is associated with the effectiveness of diuresis treatment. Furthermore, small increases in QT interval are associated with diastolic dysfunction due to impaired sarcoplasmic reticulum calcium handling as occurs in myocardial ischemia, hypertension, or diabetes. On the other hand, in patients with left ventricular dysfunction, the QRS area is associated with clinical and echocardiographic response to cardiac resynchronization therapy regardless of the type of bundle branch block. In addition, subtle ECG changes and patterns in the left bundle branch block are associated with concomitant right ventricular dilation, mostly based on the QRS axis and voltage. Notwithstanding, to identify these subtle changes in QRS require exact manual measurements that can take time. In this regard, applying artificial intelligence (AI) to the ECG can make a quicker and more complete assessment, as well as provide a low cost when applied to large populations.

**Conclusion:**

We provided an update on the evidence and clinical applications of these subtle QRS changes and patterns associated with diastolic dysfunction, reduced EF, and HF development and therapy responsiveness, as well as their applications for AI to ECG.

## Graphical abstract

The QRS duration, QRS amplitude, and QT interval are according to age and sex. Subtle changes seen in the QRS outside of the normal values according age and sex and/or the presence of abnormal QRS morphology such as QRS fragmentation can be used as an evaluation of cardiac chamber dimensions, myocardial fibrosis, low EF, RV dysfunction, diastolic dysfunction, effectiveness of diuresis treatment in HF, better selection for CRT, and arrhythmic risk.

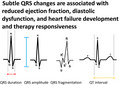

## 1 INTRODUCTION

The electrical activity of the heart was first recorded by (Lüderitz & Bayés de Luna, 2017). In the nineteenth century Willem Einthoven by using differential equations, he could predict the correct form of the human electrocardiogram (ECG) and proved his findings in 1902 using a string galvanometer, the first practical ECG (Lüderitz & Bayés de Luna, 2017).

Classification systems for ECG coding have been developed such as the Minessota code (Kors et al., 1996), as well many recommendations for interpretation of the ECG such as the scientific statements of the American Heart Association/American College of Cardiology/Heart Rhythm Society (AHA/ACCF/HRS; Rautaharju et al., 2009; Surawicz et al., 2009). The ECG is mainly used for the diagnosis of arrhythmias, intraventricular conduction disturbances, and myocardial ischemia. However, other miscellaneous diagnoses can also be made such as electrolyte abnormalities, myopericardial diseases, some genetic cardiomyopathies and channelopathies as Long QT syndrome (LQTS), Brugada syndrome, and arrhythmogenic right ventricular cardiomyopathy. Therefore, the ECG remains the noninvasive test most easily accessible, feasible, and inexpensive for cardiology assessment. In recent years, many novel ECG patterns have been published that allow for more advanced evaluation of what is currently being done, we provided and update review on the evidence of the QRS patterns associated with reduced ejection fraction, diastolic dysfunction, and heart failure therapy responsiveness.

## 2 QRS DURATION

Small increases in QRS duration are associated with a reduction of LVEF (Left ventricular ejection fraction) and increased cardiac chamber dimensions. Murkofsky et al. (1998) showed correlations of QRS duration with LV (Left ventricular) end‐systolic (*r* = .54), LV end‐diastolic counts (*r* = .54), and LVEF (*r* = −.36), QRS duration >100 ms had a sensitivity 43.8% and specificity 83.6% for the prediction of abnormal LVEF, also used an R‐wave score system defined as the sum of the R‐waves in mV in leads aVL, aVF, and V1‐V6 with cutoff value <4 mV for abnormal LVEF that correlated with LVEF (*r* = .30) and did not correlate with LV dimensions (Murkofsky et al., 1998). Similarly, the Framingham Heart Study revealed that patients with QRS between 100–119 ms compared to those with normal QRS defined as QRS < 100 ms had increased LV mass, LV diastolic dimension, and decrease systolic function in both genders, when patients with BBB (Bundle branch block) QRS ≥ 120 ms were compared with patients with normal QRS, LBBB (Left bundle branch block) was associated with increased LV mass, LV diastolic dimension, and decreased systolic function in both genders, RBBB (Right bundle branch block) was associated with increased LV mass only in women, and indeterminate BBB was associated with increased LV mass in both genders (Dhingra et al., 2005). In addition, De Winter et al. (2006) showed that QRS duration correlated with LV end‐diastolic volume (*r* = .31) and LV end‐systolic volume (*r* = .30; De Winter et al., 2006). In the MESA (Multi‐Ethnic Study of Atherosclerosis) study patients with QRS >100 ms had a risk for incident HF (Heart failure) with a HR (Hazard ratio) 2.64, 95% CI (Confidence interval), 1.66–4.2, *p* < .0001 (Ilkhanoff et al., 2012). Furthermore, Shah et al. (2016) demonstrated in patients with NSTEMI (Non‐ST‐elevation myocardial infarction) a QRS ≥ 90 ms at presentation had lower LVEF, higher severe LV dysfunction at baseline and was associated with LVEF ≤35% on follow‐up with an odds ratio (OR) 2.7, 95% CI, 5.5–4.69, *p* < .001. Additionally, a subanalysis of the MESA Study, one of the multivariable associated with the development of HFrEF (Heart failure with reduced ejection fraction) was QRS > 100 ms (HR: 2.14, 95%CI, 1.48–3.09, *p* < .001; O'Neal et al., 2017).

## 3 QRS FRAGMENTATION

The fragmentation of the QRS complex is associated with myocardial fibrosis. Flowers et al. (1969) studied the notching and slurring of the QRS complex with the results of postmortem dissections of their hearts, showed a correlation with myocardial infarction and with ventricular enlargement without scarring (Flowers et al., 1969). Thereafter, Das et al. (2006) showed in patients with QRS < 120 ms that underwent cardiac gated SPECT (Single‐photon emission computed tomography), those with fragmented QRS (f‐QRS) had higher sum wall motion score and lower LVEF. Also, f‐QRS was a more sensitive sign for myocardial scar than Q wave (sensitivity 85.6% vs. 36.3%), fragmented QRS was defined as the presence of an additional R‐wave (R') or notching in the nadir of the S‐wave, or the presence of >1 R' (fragmentation) in 2 contiguous leads, corresponding to a major coronary artery territory (Anterior leads V1 to V5, lateral leads I, aVL, V6, and inferior leads II, III, aVF) (Das et al., 2006). Moreover, a study in patients with wide QRS (QRS ≥ 120 ms) due to BBB, paced rhythms or PVC (premature ventricular complex), showed that patients with wide f‐QRS had a higher myocardial scar, total coronary occlusion, and lower EF (ejection fraction), wide‐f‐QRS was defined as the presence of an additional R‐wave (R') or notching in the nadir of the S‐wave, or the presence of >1 R' fragmentation (Das et al., 2008). The f‐QRS is associated to arrhythmic events, in patients with CAD (coronary artery disease) and nonischemic DCM (Dilated Cardiomyopathy) who received ICD (Implantable cardioverter‐defibrillator), narrow f‐QRS compared to narrow non‐f‐QRS had higher QRS duration and lower LVEF, narrow f‐QRS had higher ICD shocks, and VT (ventricular tachycardia) storm than narrow non‐f‐QRS and wide non‐f‐QRS (Das et al., 2010). Similarly, another study showed that f‐QRS in patients with Brugada syndrome are associated with recurrent VT, ICD therapy, and higher SCN5A mutations. Brugada syndrome f‐QRS was defined as ≥4 spikes in 1 right precordial lead or ≥8 spikes in all the leads V1–V3 (Morita et al., 2008). Therefore, the f‐QRS is related to myocardial fibrosis that is a substrate for the development of arrhythmic events, when the f‐QRS have a coronary artery territory distribution in the ECG is suggestive of ischemic etiology. On the other hand, Yooprasert et al. (2020) not only showed that f‐QRS was associated with myocardial scar detected by late gadolinium‐enhanced magnetic resonance imaging (MRI) sequence (OR: 2.55, CI: 1.06–6.12, *p* = .037) but also demonstrated the association between f‐QRS and ischemic heart disease (IHD) diagnosed by positive stress MRI (OR: 1.61, CI: 1.14–2.27, *p* = .007). After using multivariate analysis adjusted for age, diabetes, hypertension, renal function, and LVEF, f‐QRS remained an independent predictor for positive stress MRI (OR: 1.71, CI: 1.18–2.47, *p* = .004; Yooprasert et al., 2020). Hence, the presence of f‐QRS on standard 12‐lead ECG in the setting of patients with cardiovascular risk factors or clinical suspicion of CAD should receive a careful assessment due to the increase in the likelihood of having IHD.

## 4 QRS VOLTAGE

The low amplitude QRS voltage had been linked to pericardial diseases, infiltrative diseases, respiratory disorders with airflow limitation, advanced cardiomyopathy ischemic and nonischemic. In addition, in the last years several studies showed that it is also associated with congestive HF. Madias et al. (2001) revealed that patients with anasarca had an attenuation of the total 12‐lead QRS voltage at admission, with a peak weight gain of 48.9 ± 28.1 lbs there was a reduction of 54.2 ± 15.4% in the total 12‐lead QRS voltage compared to the admission ECG. Total 12‐lead QRS voltage correlates with weight gain (*r* = .61, *p* = .0005), the patients who subsequently lost weight increased the total 12‐lead QRS voltage (Madias et al., 2001). This suggests that for each weight gain of 7.477 lbs (3.391 kg) there was approximately a reduction of 1 mV in the total 12‐lead QRS voltage. Similarly, Lumlertgul et al. (2009) in patients with acute decompensated congestive HF compared their ECG at admission and discharge, showing after weight loss an increase in the voltage of the QRS complexes, the best correlation was with the sum QRS voltage of the six limb leads (*r* = .68, *p* = .001) and the sum of leads I and II (*r* = .55, *p* = .012). Therefore, using the sum QRS voltage of leads I and II constitute a reliable, easy, and more practical than sum all 12‐lead for assessing the effectiveness of diuresis treatment in HF (Lumlertgul et al., 2009). Furthermore, Kamath et al. used a cutoff value of <12 mV of the 12‐lead voltage for the definition of low voltage in patients with severely depressed LVEF, low QRS voltage compared to those without low QRS voltage had higher ischemic cardiomyopathy, NYHA (New York Heart Association) class 4, and atrial fibrillation. Patients with low voltage and pericardial effusion compared to those without pericardial effusion, there was no significant difference. Patients who reached the endpoint (death or HF hospitalization) compared to those who not had lower total 12‐lead voltage (Kamath et al., 2006). Therefore, the cardiac effects of increased diastolic LV volumes or pressures may lead to a decrease in the QRS voltage, and with the emergence of peripheral edema, there is a further decrease in the QRS voltage in the ECG (Madias, 2009). Besides, the amplitude of P‐waves, T‐waves, and pacemaker stimulus spikes decreases in anasarca (Madias, 2004a, 2004b, 2007). In contrast, high QRS voltage values are related to increased cardiac mass. Roberts et al. (2013) studied the QRS voltage and their relation to cardiac weight at necropsy, using a cutoff value of >17.5 mV for the sum of the total 12‐lead QRS voltage (1 mV = 10 mm), was associated to increased cardiac mass in 94% of the patients with aortic stenosis and 90% of patients with aortic regurgitation, but is not related to patients with increased cardiac mass due to cardiac amyloidosis (Roberts et al., 2013). Hence, high QRS voltage is related to increased cardiac mass secondary to valvular heart disease and some cardiomyopathies at early stages, but not to infiltrative diseases.

## 5 HEART FAILURE

In the MESA study, participants who were free of cardiovascular disease (*n* = 6664), a competing risk analysis was used to compare the association of several baseline ECG predictors with HF detected during a median follow‐up of 12.1 years. The development of HFrEF was associated with QRS >100 ms, time to intrinsicoid deflection >50 ms (Time to peak R‐wave in V5 and V6), left‐axis deviation −30°, right‐axis deviation −90°, prolonged QT interval, abnormal QRS‐T axis (woman > 77°, men > 88°), LVH (Left ventricular hypertrophy) using Cornell criteria (R AVL + S V3 ≥ 2.8 mV men, ≥2.0 mV woman), ST/T‐wave abnormalities, and LBBB. The development of HFpEF (Heart failure with preserved ejection fraction) was associated with heart rate per 10‐ms increase, abnormal p‐wave axis (outside the range of 0° and 75°), abnormal QRS‐T axis. The risk of HFrEF versus HFpEF was differently for delayed intrisicoid deflection, prolonged QT interval and ST/T‐wave abnormalities (O'Neal et al., 2017).

In the presence of LBBB, some ECG patterns that are associated with RV (Right ventricular) dilation/dysfunction had been published in recent years. Josephson et al. in the presence of LBBB, the direction of the frontal QRS axis gives information on LV activation and size, as well on the RV. Left‐axis deviation indicates a more delay in activation suggesting LV dilation and a superior axis deviation indicates additional RV dilation (Josephson & Wellens, 2015). Similarly, Van Bommel et al. (2011) demonstrated three criteria for RV dilation in patients with LBBB and severe depressed LVEF. The criteria were defined as the presence of terminal positivity in lead aVR (late R‐wave in lead aVR), low voltage in all limb leads (<0.6 mV) with normal or increased voltage in precordial leads and R/S ratio <1 in lead V5. Any combination of 2 to 3 positive criteria could predict RV dilation with a sensitivity of 81% and specificity 93% (Van Bommel et al., 2011). Moreover, (Van Stipdonk et al., 2018) showed in patients with LBBB and severe LV dysfunction the QRS area (sum of the area under the QRS complex in the calculated vectorcardiographic X, Y, and Z lead) is associated to clinical and echocardiographic response to cardiac resynchronization therapy (CRT). Identification of echocardiographic responders was better with QRS area than with QRS morphology or duration, area under the curve (AUC) 0.69 versus 0.58 and 0.58 (respectively; *p* < .001; Van Stipdonk et al., 2018).

## 6 DIASTOLIC DYSFUNCTION

QT interval is measured from the beginning of the QRS complex to the end of the T‐wave and approximates the time it takes the ventricles to repolarize. LQTS is the prolongation of the QT interval. LQTS may be either congenital or acquired (Drew et al., 2010). The National Health and Nutrition Examination Survey III (*n* = 8561) showed that risk factors for acquired prolonged QTc interval were the age, female sex, hypocalcemia, hypokalemia, history of thyroid disease, history of myocardial infarction and taking QT‐prolonging medications (Benoit et al., 2005). LQTS has been traditionally considered as purely electrical, but mechanical alterations in patients with LQTS were reported. Leren et al. (2015) Showed in patients with genotyped LQTS compared with healthy controls had a lower systolic function by global longitudinal strain (GLS), longer contraction duration and reduced diastolic function. Disturbed diastolic function occurs with impaired sarcoplasmic reticulum calcium handling as occurs in myocardial ischemia, hypertension or diabetes (Hasenfuss et al., 1999). Thus, the delayed uptake of calcium is pathophysiologically associated with prolongation of QT interval (Vyas et al., 2008). Wilcox et al. (2011) Showed that QTc was inversely associated with tissue Doppler echocardiography (TDE) e' velocity (*r* = −.54, *p* < .0001), using a cutoff value of QTc ≥ 435 ms had a sensitivity 73% and specificity 74% for detection of diastolic dysfunction, when restricted to patients with known or suspected HF had a sensitivity 80% and specificity 90%.

Another ECG pattern associated with diastolic dysfunction is the T‐peak to T‐end interval (TpTe). Sauer et al. (2012) demonstrated that increased TpTe was inversely associated with reduced TDE e' velocity (*R* = −0.66, *p* < .0001) and used a cutoff value of TpTe ≥ 75 ms for detection of abnormal diastolic stress test (Peak exercise E/e' > 13). Moreover, Namdar et al. (2013) showed that T‐peak to P‐wave (Tend‐P) interval using a cutoff value of ≤311 ms had a sensitivity 79% and specificity 72% for detection of diastolic dysfunction, but using a novel index calculated as Tend‐P interval/PQ interval x Age using a cutoff value of <0.0333 had an AUC 0.91, sensitivity 82% and specificity 93% for detection of diastolic dysfunction (Namdar et al., 2013). A retrospective study (*n* = 204) of patients without coronary artery disease, reported that using this formula [aVL R amplitude × (V1S amplitude + V5R amplitude)/D1 P amplitude] can predict diastolic dysfunction in echocardiography. A ROC analysis showed that the optimal cutoff value to predict diastolic dysfunction was 8.53 mV with 70% sensitivity and 70% specificity (AUC: 0.78; 95% CI: 0.71–0.84, *p* < .001; Hayıroğlu et al., 2021).

## 7 ARTIFICIAL INTELLIGENCE APPLICATIONS IN ECG

Neural networks are machine learning models inspired by the organization of the human brain. A neuron in the hidden layer is activated when input neurons pass a large enough value to trigger the neuron, much like a biological neuron. Activated neurons continue to pass a value to the next layer of neurons until the final “output layer” of neurons is reached. Deep learning is a powerful method premised on learning complex hierarchical representations from the data that constitute multiple levels of abstraction. The earliest application of neural networks in medicine dates to at least 1995. Since then, remarkable progress has been made with the Artificial Intelligence (AI) in the medical field, with the use of machine learning techniques as the deep convolutional neural networks (Johnson et al., 2018). For example, in dermatology classifying skin photographs into malignant melanomas versus benign nevi with a level of competence comparable to dermatologists (Esteva et al., 2017), or in ophthalmology for detection of diabetic retinopathy and diabetic macular edema in retinal fundus (Gulshan et al., 2016). Similarly, in Cardiology, the AI had been applied with speckle‐tracking echocardiographic databases for discrimination of physiological versus pathological patterns of hypertrophic remodeling (Narula et al., 2016). Moreover, AI had been applied to a noninvasive acoustic device to analyze the frequency range and timing of microbruits waves originating from blood flow turbulence in the coronary circulation during diastole. Recent advances in sound sensor technology, analytic power and data filtering have enabled the use of acoustic detection to diagnose intracoronary turbulence due to obstructive CAD. These noninvasive acoustic devices have a negative predictive value (NPV) of 96% and positive predictive value (PPV) of 16%, (Winther et al., 2018). In the field of ECG, recently had come out some studies with AI application to ECG for detection of low EF with an AUC 0.93, sensitivity 86.3%, specificity 85.7%, these AI was trained by feeding it raw data from ECGs accompanied by information on LVEF, hence AI can detect these subtle patterns that human is unaware (Attia, Kapa, et al., 2019). It is well known that Sex and age have long been known to affect the ECG. QT interval, QTc interval and nonspecific ST‐segment deviation is greater in women than men, whereas QRS amplitude and QRS is larger in males than females. Probably as a result of several biologic variables such as hormones, and anatomic factors such as body mass index may contribute to sex and age‐related differences on the ECG. A study using AI to the ECG showed 90.4% classification accuracy with an area under the curve of 0.97 for sex classification. Age was estimated as a continuous variable with an average error of 6.9 ± 5.6 years (*R*‐squared = 0.7). Major factors seen among patients with a neural networks‐predicted age that exceeded chronologic age by >7 years included: low EF, hypertension, and coronary artery disease (CAD) *p* < .01 (Attia, Friedman, et al., 2019). A study showed that ECG‐based detection of hypertrophic cardiomyopathy (HCM) by an AI algorithm has an AUC of 0.96 (95% CI: 0.95–0.96) with sensitivity 87% and specificity 90%. In subgroup analyses, the AI algorithm achieved with high diagnostic performance, particularly in younger patients (sensitivity 95%, specificity 92%). Nevertheless, this model requires further refinement and external validation, but it may hold promise for HCM screening (Ko et al., 2020). Another study with AI algorithm using only 2 ECG leads, a deep‐learning model detected hyperkalemia in patients with renal disease showed a high sensitivity with a NPV 99% and low specificity PPV 8.7 to 14.8, with an AUC of 0.853 to 0.883. The application of artificial intelligence to the ECG may enable screening for rule out hyperkalemia (Galloway et al., 2019). Similarly, another study demonstrated the detection of LV diastolic dysfunction with an AUC 0.91, sensitivity 80%, specificity 84% and, also correctly identified concomitant significant CAD in 82% (Sengupta et al., 2018). On the other hand, HFpEF is defined by LVEF ≥ 50% and the dominant abnormality resides in diastole (Borlaug, 2014), In addition, a study demonstrated in patients with HFpEF the 68 percent had angiographically proven CAD (Hwang et al., 2014). Therefore, the use of AI application to ECG could be a potential tool for the diagnosis of CAD in together with noninvasive tests.

## 8 CONCLUSION

Subtle changes seen in the QRS can be used as an evaluation of cardiac chamber dimensions, myocardial fibrosis, low EF, RV dysfunction, diastolic dysfunction, effectiveness of diuresis treatment in HF, better selection for CRT, and arrhythmic risk (Table 1). Many physicians are unaware of the importance of the QRS. Notwithstanding, to identify these subtle changes in QRS require exact manual measurements that can take time. Hence, the application of AI to the ECG can make a quicker and more complete assessment, as well providing a low cost when is applied to large populations.

**TABLE 1:** Studies related to ECG patterns associated with reduced ejection fraction, diastolic dysfunction, and heart failure

Author/year	ECG interpretation	Study method and setting	Results and conclusion
Murkofsky et al. (1998)	QRS > 100 ms is an indicator of LVEF < 45%	226 consecutive patients referred to a nuclear cardiology laboratory. QRS measuring and calculation of the R‐wave scoring system (sum of R‐waves in mV in leads aVL, aVF and V1‐V6)	For each successive 0.001‐s ↑ in the definition of prolonged QRS duration (from >0.10 to >0.12 s) there was an ↑ in SP from 83.6% to 99.3%, with ↓ in SE from 43.8% to 13.8% for prediction of LVEF <45%. An R‐wave score < 4 mV had a 33 SE and a 95% SP for the prediction of LVEF <45%
Madias et al. (2001)	For each weight gain of 7.477 there was a rough reduction of 1 mV in the total 12‐lead QRS voltage	28 patients admitted to the coronary care unit with anasarca. Measure the sums of the amplitude of QRS complexes and correlation with weight	Attenuation of QRS voltage in patients with anasarca correlates with weight gain (*r* = .61, *p* = .0005). Pericardial effusions were excluded
Dhingra et al. (2005)	↑ QRS duration (≥100 ms) was related to increase LV mass, LV dimensions and decrease systolic function	4534 participants of the Framingham Heart Study. QRS duration and echocardiographic LV dimensions Excluded prevalent HF, previous MI, pacemaker and digoxin or quinidine use	LBBB was associated with higher LV mass, LVDD, and with lower FS compared with the referent group (QRS < 100 ms) in both genders. RBBB was not related to any LV measurement in men. RBBB was associated with LV mass and wall thickness in women. Indeterminate BBB was related to LV mass and wall thickness in both genders
De Winter et al. (2006)	QRS ≥ 120 ms is associated with ↑ LV end‐diastolic and LV end‐systolic volume	132 consecutive patients with CAD and mean LVEF 24 ± 6. Perfusion SPECT study using technetium‐99 m was used to obtain resting LVEF and volumes	Patients with QRS ≥ 120 ms compared to patients with QRS < 120 ms had higher LV end‐diastolic volumes (248 ± 77 vs. 205 ± 73, *p* < .01) and LV end‐systolic volumes (193 ± 68 vs. 159 ± 60, *p* < .01)
Das et al. (2006)	f‐QRS with a QRS < 120 ms in 2 leads corresponding to coronary artery territory had SE 85.6% and SP 89.4% for myocardial scar detected by SPECT	479 consecutive patients for cardiac gated SPECT. The f‐QRS was defined by the presence of an additional R‐wave or notching in the nadir of the S‐wave, or the presence of >1 R´ in 2 leads, corresponding to a coronary artery territory (anterior V1 to V5, lateral I, aVL, V6, and inferior II, III, aVF)	ECG sign for myocardial scar: Q wave had SE: 36.3%, SP: 99.2%, PPV: 95.7% and NPV: 70% f‐QRS had SE: 85.6%, SP: 89.4%, PPV: 83.7%, NPV: 87.6 f‐QRS and/or Q wave had SE: 91.4%, SP: 89%, PPV: 84.2% and NPV: 94.2% The ROC curves of ECG sign for myocardial scar: f‐QRS 0.82(95% CI: 0.78–0.86) and q wave 0.65 (95% CI: 0.59–0.70), *p* < .001
Kamath et al. (2006)	Sum of total 12 lead QRS voltage <12 mV is a marker of worse functional class and a risk factor for death and HF hospitalization in patients with systolic HF at 1 year	415 patients with severely depressed LVEF followed up for 1 year, endpoint death and hospitalization for HF (HF clinic cohort 1), 100 patients with advanced HF who had an ECG within 1‐year preceding cardiac transplantation (pretransplant cohort 2). Low voltage was defined as the total 12 lead ECG voltage <12 mV	Low voltage was associated with more ischemic cardiomyopathy (53 vs. 32, *p* < .001), NYHA class 4 (23 vs. 14, *p* = .04), LV end‐diastolic dimension (6 ± 0.9 vs. 6.3 ± 0.9, *p* = .03), LV end‐systolic dimension (5 ± 1 vs. 5.4 ± 0.9, *p* = .005). Patients who reach the endpoint (*n* = 108) compared to those who not (*n* = 307) had lower total 12‐lead voltage (14 ± 5 vs. 16 ± 6, *p* < .001), Patients classified as a low voltage by 12‐lead <12 mV had ↑ risk of death (18% vs. 7%, *p* = .008), ↑risk of death or HF hospitalization (34% vs. 22, *p* = .04)
Das et al. (2008)	The fw‐QRS with a QRS ≥ 120 ms (BBB, pacedQRS or PVC) in 2 leads corresponding to a coronary artery territory had SE 86.8% and SP 92.5% for myocardial scar detected by nuclear stress imaging	879 patients underwent to cathlab or gated SPECT. F‐BBB defined as various RSR' patterns with or without a Q wave, with 2 > R‐waves (R´) or >2 notches in the R‐wave, or >2 notches in the downstroke or upstroke of the S‐wave, in 2 leads corresponding to a coronary artery territory. F‐PVC defined by >2 R'or >2 notches in the S‐waves in 2 contiguous leads. The f‐PVC also included PVCs with only 2 notches in the R‐wave but were > 40 ms apart and presented in 2 contiguous leads. f‐pQRS defined by >2 R'or > notches in the S‐waves in 2 contiguous leads and without any evidence of QRS fusion	Patients with f‐wQRS (*n* = 415) compared to without f‐wQRS (*n* = 464) had higher myocardial scar (92% vs. 12.5%, *p* < .001), death (35.9% vs. 18.1, *p* < .001) and lower ejection fraction % (38 ± 15 vs. 49 ± 14, *p* < .001). Kaplan–Meier survival analysis revealed ↑mortality in the f‐wQRS group when compared with the wQRS group (*p* < .001) mean follow‐up 29 ± 18 months. The subgroup analysis also revealed that fragmented compared to nonfragmented subgroups had ↓ time to death: f‐BBB vs. BBB, *p* = .05. f‐PVC vs. PVC, *p* = .001. f‐pQRS vs. pQRS, *p* = .008. Further analysis of the f‐BBB group revealed that f‐LBBB but no f‐RBBB was associated with ↓ time to death compared with LBBB (*p* = .003) and RBBB (*p* = .88). f‐wQRS sign for myocardial scar had a SE 86.8 (95% CI, 83.6–90), SP 92.5 (95% CI, 90–95), PPV 92 (95% CI, 89.4–94.7) and NPV 87.5 (95% CI, 84.5–90.5)
Morita et al. (2008)	The f‐QRS ≥ 4 spikes in 1 right precordial lead or ≥8 spikes in all the leads (V1–V3) is a marker for spontaneous VF and predicts recurrent syncope in patients with Brugada syndrome	115 patients with Brugada syndrome (*n* = 13 resuscitated from VF, *n* = 28 syncope, *n* = 74 asymptomatic). Assess f‐QRS in the right precordial leads. Follow‐up 43 ± 25 months	The f‐QRS was observed in 43% (*n* = 50) of the patients, more often in VF group (VF 85% [11/13], syncope 50% [14/28] and less in asymptomatic 34% [25/74], *p* = .0069) SCN5A mutations were higher in the f‐QRS group (34%) than patients without f‐QRS (5%) *p* = .0027 EPS: Patients with f‐QRS vs. patients without f‐QRS had a longer RR interval (899 ± 125 vs. 986 ± 226 ms, *p* = .0087) and longer HV interval (39 ± 7 vs. 44 ± 7 ms, *p* = .0015). Outcome: Recurrent VF 15(30%) vs. 1(2%) *p* < .001. ICD therapy 28 (56%) vs. 12 (18%) *p* = .006
Lumlertgul et al. (2009)	Good correlation between the weight loss and the increase in the sums of QRS voltage of leads I and II in patients with congestive HF	20 consecutive patients with congestive HF admitted for acute decompensated heart failure. ECGs and weights recorded on admission and at discharge. QRS complexes in all ECG leads were measured, four sets of ECG leads measurement	Admission and discharge ECG measurements: QRS 12 leads 136.50 ± 45.85, 151.83 ± 41.43, *p* = .003 QRS precordial 105.52 ± 43.26, 118.17 ± 39.11, *p* = .006 QRS limb leads 31.55 ± 9.63, 36 ± 10.01, *p* = .0002 QRS 2 leads (I and II) 11.3 ± 4.17, 13.06 ± 4.1, *p* = .001 Correlation of weight %Δ (change) in QRS: 12 (*r* = .192, *p* = .404), QRS precordial (*r* = .036, *p* = .845), QRS limb (*r* = .679, *p* = .001) and QRS lead I and II (*r* = .552, *p* = .012)
Das et al. (2010)	The f‐wQRS (QRS < 120 ms) in 2 contiguous leads corresponding to a major coronary artery territory is a predictor of arrhythmic events	361 patients with CAD (*n* = 245) and DCM (*n* = 116) who received ICD for primary or secondary prevention of SCD. Mean follow‐up 16.6 ± 10.2 months. Assess arrhythmic event and mortality. VT storm defined as ≥ ICD shocks in 24 h	The f‐QRS group vs. non‐f‐QRS had ↑ ICD shock (37% vs. 10%, *p* < .001), ATP (19.1% vs. 3%, *p* < .001), VT storm (13.1% vs. 2%, *p* = .007) and no difference in death (13.1% vs. 8%, *p* = .33). f‐QRS group vs. QRS ≥ 120 ms group (*n* = 177) had ↑ ICD shock (37% vs. 23%, *p* < .001), ATP (19.1% vs. 10.2%, *p* = .03), VT storm (13.1% vs. 9%, *p* = .02), wQRS group had ↑ death (13.1% vs. 19.8%, *p* = .009).
Wilcox et al. (2011)	QTc ≥435 ms had sensitivity 73% and specificity 74% for detection of DD, when restricted to patients with known or suspected HF sensitivity 80% and specificity 90%	Study 1: 75 patients referred for clinical suspicion of HF. Study 2: 100 unselected patients undergoing echocardiography. Graded DD as: Normal (septal E' > 8 cm/s), grade I DD (septal E' < 8 cm/s and E/A ratio < 0.8), grade II DD (septal E' < 8 cm/s and E/A ratio 0.8–1.5), grade III DD (septal E' < 8 cm/s and E/A ratio > 1.5 or E deceleration time < 150 ms)	Study 1: QTc was inversely associated with E' velocity (*r* = −.54, *p* < .0001), area under the receiver operating characteristic curve for QTc as a predictor of DD was 0.82 QTc ≥ 435 ms had SE: 73% and SP: 74% for detection of DD. Per each standard deviation ↑ in the QTc interval, there was a 4‐fold ↑ in the odds of septal E' < 8 cm/s (95% CI: 1.2–13.4, *p* = .026) and 3.7‐fold ↑ odds of an increased E/E' ratio > 15 (95% CI 1.3–10.5, *p* = .013). Study 2: The c‐statistic for QTc as a predictor of septal E' < 8 cm/s was 0.62. Cutoff for QTc >435 ms had SE 80% and SP 90% when suspected HF
Van Bommel et al. (2011)	Criteria for RV dilation in LBBB: Terminal positivity in lead aVR (late R‐wave in lead aVR). Low voltage (<0.6 mV) in all extremity leads with normal or increased voltage in precordial leads R/S ratio < 1 in V5	173 patients with heart failure (52% nonischemic) with a mean LVEF 24 ± 8 and known LBBB. ECG and Echocardiographic assessment of RV dimensions. RV dilation defined as an RV base‐to‐apex length ≥ 86 mm or an RV diastolic area ≥ 33 cm^2^	RV base‐to‐apex length ≥ 86 mm was present in 39% (*n* = 67) and RV diastolic area ≥ 33 cm^2^ in 36% (*n* = 62) Patients with 2 or 3 positive criteria compared to patients with 0 positive criterion had higher RV base‐to‐apex length (90 ± 5 vs. 78 ± 6, *p* < .001), RV diastolic area (34 ± 5 vs. 25 ± 6, *p* < .001), tricuspid regurgitation grade (16 ± 4 vs. 18 ± 3, *p* = .034) and pulmonary artery systolic pressure (38 ± 7 vs. 30 ± 8, *p* < .01). 2 to 3 positive criteria were able to predict an RV base‐to‐apex length ≥ 86 mm with a sensitivity 81% and specificity 93% 2 to 3 positive criteria were able to predict an RV diastolic area ≥ 33 cm^2^ with a sensitivity 79% and specificity 89%
Ilkhanoff et al. (2012)	QRS > 100 ms is associated with incident HF and a marker of abnormal cardiac measures by cardiac imaging test	4591 participants of the Multi‐Ethnic Study of Atherosclerosis (MESA). Follow‐up of 7.1 years. 75 participants developed incident HF. Measure QRS, objective incident HF and measures of cardiac structure and function by cardiac MRI	Patients with QRS duration >100 ms had ↑ risk for incident HF (HR: 2.64, 95%, 1.66–4.2, *p* < .0001). QRS > 100 ms had a SE of 38.7%, SP: 80.7% and NPV 98.8% for predicting HF. MRI measures of the QRS > 100 ms group (*n* = 3961) vs. QRS ≤ 100 ms group (*n* = 900) had greater LVEDV 72.3 ml (95% CI, 71.4–73.2) vs. 67.5 (95% CI, 67–67.9, *p* < .01), LVESV 23.8 ml (95% CI, 23.3–24.3) vs. 21 (95% CI, 20.8–21.3, *p* < .01), LV mass 83.8 (95% CI, 82.9–84.8) vs. 77 (95% CI, 74–76.5), LVSV 48.5 (95% CI, 47.9–49‐1) vs. 46.4 (95% CI, 46.1–46.7, *p* < .01)
Sauer et al. (2012)	T‐peak to T‐end ≥75 ms in V5 is associated with resting DD and peak exercise E/e' ratio	84 consecutive unselected patients referred for exercise echocardiography, treadmill test Bruce, echocardiography assessment of DD at rest and peak stress. ECG measurement of T‐peak to T‐end (TpTe) in V4–V6, preferably in V5 (manual analysis). The patients were divided in 2 groups by median TpTe <75 ms (*n* = 41) and TpTe ≥75 ms (*n* = 43). LVEF >55% in all patients. Excluded ECG with U‐wave and T‐wave amplitude <1.5 mV	Group TpTe ≥ 75 ms (*n* = 43) compared to TpTe <75 ms (*n* = 41) had lower peak heart rate (149 ± 23 vs. 164 ± 21, *p* = .002) exercise capacity METs (11 ± 3.5 vs. 12.6 ± 3.4, *p* = .049), septal e' tissue velocity cm/s (11.2 ± 4.1 vs. 14.7 ± 5.2, *p* < .0001), and E/e' ratio > 13 (23% vs. 2%, *p* = .007). Inverse relationship between TpTe and e' velocity (*R* = −.66, *p* < .0001), TpTe and QTc (*R* = −0.28, *p* = .011). The multivariable model showed each 10‐ms increase in TpTe was associated with a 0.41 cm/s decrease in e' velocity (*p* = .006). After adjusted for age, QTc interval, LV mass index, presence of wall motion abnormality TpTe was also independently associated with worse DD (OR: 3.9, 1.4–10.7, *p* = .009)
Namdar et al. (2013)	T‐end to P (Tend‐P) ≤311 ms had a SE 79% and SP 72% for diagnosis of DD. Tend‐P/PQ x Age ≥0.0333 had a SE 82% and SP 93% for diagnosis of DD. PQ ≥150 ms had a SE 78% and SP 46% for diagnosis of DD	Derivation group: 81 patients with DD (67 DD 1 and 14 DD2) were analyzed and 83 unselected normal controls Validation group: 50 patients with DD1 and 50 normal controls T‐waves smaller than 1.5 mm in amplitude were not included in the analysis. Excluded atrial fibrillation, higher than grade 1 AV‐block, atrial and/or ventricular pacing	Calculation of Tend‐P/PQ x Age with a cutoff value of ≥0.0333 showed AUC: 0.91, SE: 82%, SP: 93%, PPV: 93% and NPV: 82% for diagnosis of DD. ‐PQ ≥ 150 ms AUC: 0.65, SE: 78%, SP: 46%, PPV: 58%, NPV: 68%. ‐Tend‐P ≤ 311 ms AUC: 0.82, SE: 79%, SP: 72%, PPV: 74%, NPV: 78%. Validation group: Patients with DD1 (*n* = 50) vs. controls (*n* = 50) had ↑ P‐wave duration ms (113 ± 21 vs. 101 ± 11, *p* < .0001), P‐wave dispersion (57 ± 18 vs. 51 ± 17, *p* < .005), QTc ms (438 ± 32 vs. 416 ± 28, *p* < .05), lower Tend‐P (237 ± 131 vs. 349 ± 152, *p* < .0005) and Tend‐Q ms (404 ± 129 vs. 494 ± 141, *p* < .0001)
Shah et al. (2016)	QRS ≥ 90 ms had lower LVEF at the time of NSTEMI and was associated with severely reduced LVEF on follow‐up	536 patients with NSTEMI. LVEF at baseline, follow‐up 12 months. Bundle branch block and paced rhythm were excluded	QRS ≥ 90 ms group compared with QRS < 90 ms group had lower LVEF (47 ± 15% vs. 50 ± 13%, *p* = .038), had higher severe LV dysfunction (LVEF≤35%) at baseline (27% vs. 18%, *p* = .045). Logistic regression analysis revealed that QRS ≥ 90 ms was independently associated with severely reduced EF ≤ 35% on follow‐up (OR = 2.7;CI: 1.55–4.69, *p* < .001)
O'Neal et al. (2017)	Risk of HFrEF vs. HFpEF was different for abnormal time to intrinsicoid deflection, prolonged QT interval, and ST/T‐wave abnormalities	6664 participants from the Multi‐Ethnic Study of Atherosclerosis (MESA) who were free of cardiovascular disease at baselines. Analysis of baseline ECG predictors with HFrEF and HFpEF detected during a median follow‐up of 12.1 years	HFrEF ECG markers: QRS > 100 ms (HR, 95% CI, 2.14, 1.48–3.09, *p* < .001), time to intrinsicoid deflection (ID) > 50 ms (HR, 95% CI, 4.90, 2.77–8.68, *p* < .001), left‐axis deviation (HR, 95% CI, 1.86, 1.15–3.02, *p* = .012), right‐axis deviation (HR, 955 CI, 4.98, 1.22–20.40, *p* = .025), prolonged QT interval (HR, 95% CI, 2.39, 1.55–3.68, *p* < .001), abnormal QRS‐T axis (HR, 95% CI, 2.52, 1.56–4.05, *p* < .001), left ventricular hypertrophy (HR, 95% CI, 2.56, 1.36–4.82, *p* = .0036), ST/T‐wave abnormalities (HR, 95% CI, 2.47, 1.69–3.62, *p* < .001) and LBBB (HR 95% CI, 6.75, 2.70–16.86, *p* < .001). HFpEF ECG markers: Heart rate per 10‐ms increase (HR, 95% CI, 1.34, 1.12–1.60, *p* = .0014), abnormal P‐wave axis (HR, 95% CI, 2.04, 1.22–3.42, *p* = .0066) and abnormal QRS‐T axis (HR, 95% CI, 2.01, 1.21–3.33, *p* = .0068)
Van Stipdonk et al. (2018)	QRS area ≥ 109 μVs is associated with echocardiographic CRT response and higher event‐free survival	1491 consecutive patients selected for CRT device implantation ECG records were stored digitally in the MUSE Cardiology information system (GE Medical System), Custom MatLab software (MathWorks Inc, Natick, MA) was used to convert the 12‐lead ECG into the 3 orthogonal vector cardiography (X, Y, and Z lead) and the QRS area was calculated. Follow‐up 3.4 ± 2.4 years. (CRT response was defined as a reduction in LVESV ≥ 15%)	Patients with QRS area ≥ 109 μVs compared with QRS area < 109 μVs had a higher event‐free survival (HR: 0.6, 0.46–0.77, *p* < .001). Patients with QRS area > 150 μVs compared with QRS area of 109–150 μVs had lower hospitalization rate (HR: 0.49, 0.34–0.71, *p* < .001). Patients without a class I indication (LBBB, QRS > 150 ms) with QRS area ≥ 109 μVs (n = 97) vs. QRS <109 μVs (*n* = 270) resulted in better response rates 54% vs. 38%, *p* < 0.001 (OR: 1.90 1.19–3.03, *p* = .009). Patients with a class I indication with QRS area ≥ 109 μVs (*n* = 377) compared with QRS <109 μVs (*n* = 147) resulted in significant separation of response rates 73% vs. 44%, *p* < .001 (OR: 3.54, 2.38–5.28, *p* < .001). Multivariable showed independent association of QRS area to LVESV reduction ≥15% (OR: 1.65, 1.43–1.9, *p* < .001). Identification of echocardiographic responders was better with QRS area than with QRS morphology or duration (AUC, 0.69 vs. 0.58 and 0.58, respectively; *p* < .001

Abbreviations: ATP, antitachycardia pacing; BMI, body mass index; DD, diastolic dysfunction; EF, ejection fraction; EDC, end‐diastolic counting; EPS, electrophysiology study; ESC, end‐systolic counting; f‐BBB, fragmented bundle branch block; f‐LBBB, fragmented LBBB; f‐RBBB, fragmented RBBB; f‐pQRS, fragmented pacedQRS; f‐PVC, fragmented premature ventricular complex; f‐wQRS, fragmented wide QRS; LAD, left anterior descending artery; LCX, left circumflex artery; LVDD, left ventricular internal dimensions in diastole; MRI, magnetic resonance imaging; NPV, negative predictive value; PPV, positive predictive value; pQRS, pacedQRS; RCA, right coronary artery; ROC, receiver operating characteristic; wQRS, wide QRS; SE, sensitivity; SP, specificity; vs., versus.

## AUTHOR CONTRIBUTION

Artemio García‐Escobar: Responsible for the concept design, writing and editing artificial intelligence applications in ECG, conclusion, table1, and graphical Abstract; Silvio Vera‐Vera: Writing and editing QRS duration and QRS fragmentation; Alfonso Jurado‐Román: Writing and editing QRS voltage; Santiago Jiménez‐Valero: Writing and editing heart failure; Guillermo Galeote: Writing and editing introduction; Raúl Moreno: Writing and editing diastolic dysfunction.

## CONFLICT OF INTEREST

None declared.

## ETHICAL STATEMENT

Not applicable.

## Data Availability

All data generated or analysed during this study are included in this review article. Any further data and material can be received through contacting the corresponding author
